# Arthroscopic Reconstruction of an Anterior Cruciate Ligament Tear Using the Anatomic Single-Bundle Technique: A Clinical and Functional Outcome Evaluation

**DOI:** 10.7759/cureus.69069

**Published:** 2024-09-10

**Authors:** Mahak Baid, Shamik Hait, Saurabh Daga, Ayon Das, Asish Kumar Mandal, Jerry Sam, Karthikeyan Dhandapani, Ali Amjad, Prashanth D'sa

**Affiliations:** 1 Orthopaedics, Aneurin Bevan University Health Board, Newport, GBR; 2 Orthopaedics, Baksi Orthopaedics Trauma and Rehabilitation Centre, Kolkata, IND; 3 Orthopaedics, University Hospitals Birmingham NHS Foundation Trust, Birmingham, GBR; 4 Orthopaedics, Employees State Insurance Post Graduate Institute of Medical Sciences and Research (ESI-PGIMSR) Employees State Insurance Corporation (ESIC) Medical College and Hospital, Kolkata, IND; 5 Orthopaedics, Iris Multispeciality Hospital, Kolkata, IND

**Keywords:** anterior cruciate ligament, arthroscopy, hamstring graft, lysholm score, single-bundle reconstruction

## Abstract

Background: The anterior cruciate ligament (ACL) consists of an anteromedial bundle and a posterolateral bundle giving anteroposterior and rotational stability to the knee. An ACL tear might lead to secondary changes in the knee joint if not operated in time. The aim of the study was to evaluate the clinical and functional results in patients with ACL tears treated by arthroscopic reconstruction using the anatomic single-bundle technique.

Methods: This was a prospective study conducted between June 2015 and December2017 at a teaching institute in Kolkata, India, on patients who underwent single-bundle arthroscopic reconstruction of an ACL tear. A minimum follow-up of nine months was considered for all patients. The functional outcome was assessed via the Lysholm knee score.

Results: A total of 45 patients were included in this study, of which 34 (75.56%) and eight (17.78%) patients showed excellent and good results, respectively, at the final follow-up. The mean age in this study was 29.88±9.02 years. No complication was seen in 95.6% of patients. The mean Lysholm score by the end of nine months was 95.31±6.55. At the time of the final follow-up, all the patients returned to their same activity status prior to injury.

Conclusion: Arthroscopic anatomic single-bundle ACL reconstruction using hamstring autograft is an effective treatment modality for ACL injuries. It restores the stability of the knee and is associated with good recovery of joint function with an early return to active lifestyle and sports activities. This procedure achieves excellent clinical and functional outcomes without any long-term disability.

## Introduction

The anterior cruciate ligament (ACL) is the main stabilizing structure of the knee joint as it maintains forward and rotational stability and prevents anterior translation of the tibia over the femur [1]. The ACL consists of two functional bundles, the anteromedial (AM) and posterolateral (PL) bundles [2], which are named anatomically after their position of attachment on the tibia. Biomechanically, these two bundles function together to provide stability to the knee throughout the range of motion (ROM) [3]. The AM bundle is primarily responsible for knee stabilization in the anteroposterior direction, whereas the PL bundle provides rotational stability [4]. The AM bundle remains tight in flexion, whereas the PL bundle is tight during extension [5].

The ACL is one of the most commonly injured ligaments of the knee. About 70% of ACL injuries occur through non-contact mechanisms such as sudden deceleration, cutting maneuvers involving a quick change of direction, or landing improperly, while 30% result from direct contact usually as a result of a valgus force acting on the knee [6].

With the recognition of ACL tears came the consensus that untreated ACL injuries often lead to progressive deterioration of the knee with damage to menisci and cartilage. The healing response after ACL rupture is poor [7], and thus, without surgical reconstruction, the function of ACL-deficient knee is limited. Future degenerative changes and premature arthritis of the knee can occur in patients not opting for the surgical management of an ACL tear.

More than 400 different techniques for ACL reconstruction have been described to date from open to arthroscopic methods [8]. Arthroscopic ACL reconstruction has the advantages of being minimally invasive, having a more accurate placement of the graft, and having quicker recovery and rehabilitation with minimal hospital stay. ACL reconstruction has been reported to have a success rate of 83-95% [9].

Anatomic ACL reconstruction is the restoration of the ACL to its native dimensions, collagen orientation, and insertion sites [10]. A single-bundle (SB) ACL reconstruction recreates the AM bundle and ignores the PL bundle in comparison to the double-bundle (DB) reconstruction.

In recent times, the DB reconstruction technique has gained popularity. However, it requires a longer operative time, is technically more demanding, involves an increased risk of graft impingement and lateral femoral condyle and bone bridge fractures, is challenging in revision surgery [11], and has limited evidence of superior results when compared to the SB technique [12].

The centrally placed anatomic SB ACL reconstruction is a common operative procedure and has been proven to restore normal knee function [13,14]. Moreover, SB reconstruction is still the most frequently performed method and is also useful when DB reconstruction cannot be performed such as in cases with open growth plates, severe arthritic changes, multiple ligament injuries, a narrow notch, severe lateral femoral condyle bone bruising, or tear of only one ACL bundle [15].

The objective of this study was to depict our experience constituting cases of ACL tears which were reconstructed by arthroscopic SB technique to see whether this modality of management achieves stable knees, good ROM, faster recovery, and early return to daily activities with enhanced clinical and functional outcome in the Indian scenario.

## Materials and methods

This was a prospective study in which 45 patients with ACL tears who were treated by arthroscopic reconstruction using the SB technique in the Department of Orthopaedics, Medica Superspecialty Hospital, Kolkata, India, from June 2015 to December 2017 and fulfilling the inclusion criteria were considered for the study. Approval was obtained from the Clinical Research Ethics Committee of Medica Superspecialty Hospital (approval number: CREC/2015/July/07).

Inclusion criteria

Criteria for inclusion were as follows: 18-50 years of age, chronic injury of more than three weeks duration, and radiologically established complete ACL tear with clinical instability.

Exclusion criteria

Exclusion criteria were the following: associated other ligament or meniscus injury of the same joint, clinicoradiologically established arthritis of the knee joint, previous bony fracture of the same limb, and history of prior surgery of the affected knee joint.

Operative procedure

A thorough history of the patients was taken, clinical examination was performed, and preoperative routine laboratory investigations were done. These were supplemented by X-rays in anteroposterior and lateral views along with an MRI of the knee joint.

Spinal anesthesia was done in all patients. They were placed in a supine position with the knee in 90° flexion. A pneumatic tourniquet was used in all cases. Arthroscopy of the knee joint was done through a high anterolateral viewing portal, anteromedial instrumental portal, and accessory medial instrumental portals. Diagnostic roundups were done to evaluate the intra-articular abnormalities like chondral defect, meniscal tear, loose bodies, and confirmation of ACL tear pattern.

The semitendinosus tendon was harvested and divided into two units of equal appropriate size, and Krackow sutures were applied at both ends of each unit with a fiber wire, and then they were quadrupled over the Arthrex TightRope loop device. The free ends of the tendon graft were sutured at the non-looped end using a vicryl suture. The graft was passed through a series of calibrated cylinders in the graft sizing block to determine the femoral and tibial tunnel diameter. The four threads of fiber wire from each side were then passed into each of the holes of the suture disc.

With Steadman awl, a hole was punched at a point 5-7 mm from the posterior edge of the lateral femoral condyle at the 10 o'clock position of the right knee and the two o'clock position of the left knee. The femoral tunnel was drilled using the 4 mm drill tip graduated guide pin. The point of the cortical break was noted on the pin which gave us the length between the lateral femoral cortex and the inner surface of the lateral femoral condyle. Keeping the knee in hyperflexion, the drill guide was passed out through the skin on the lateral thigh. An appropriate size reamer according to the precise diameter of the graft was inserted over the guide pin through the accessory medial portal, and the femoral tunnel was reamed up to 2 cm from the inner cortex of the lateral femoral condyle at the point of entry of the guide pin. A looped end of the fiber wire was passed into the eye of the guide pin, and the free end was drawn out of the lateral part of the thigh.

Now, a Director ACL Tip Aimer (50°) was inserted through the accessory medial portal keeping the knee at 90° of flexion. The midpoint of the tibial stump of the native ACL was identified, and a 2.4 mm guide pin was drilled into the tibia after emplacing it marginally medial to the center of the intercondylar region. Arthroscopic confirmation of the guide pin's position was done. Then drilling of the tibial socket was carried out keeping it proportionate to the distal diameter of the ACL graft.

The suture loop was retrieved into the tibial tunnel by using a grasper. This loop was passed through the femoral tunnel and was left hanging out of the accessory medial portal. The lead sutures (white and green) of the TightRope were passed through the suture loop and were put out of the thigh laterally. Using the sutures tied to the TightRope EndoButton, the ACL graft was then tugged into the knee. The green suture was pulled to slide the EndoButton longitudinally into the femoral tunnel up to the premeasured and marked length reading of the femoral pin initially.

After the premarked loop length enters through the femoral tunnel under arthroscopic vision, the white thread of the EndoButton coming out of the lateral thigh is pulled out. This flips the EndoButton on the posterolateral cortex of the femur. The green thread was pulled out, and the white threads were used to toggle the EndoButton and tighten the TightRope pulling the premeasured 2 cm of graft into the femoral tunnel. Intra-articular marking of the graft at 2 cm was identified and confirmed. Tibial site fixation was done at 30° flexion of the knee by tying the four threads over the suture disc (14 mm) on the cortex of the tibial tunnel entry. The graft was then tensioned by toggling at the femoral end to remove the remaining laxity.

Surgical portals were closed in layers. A sterile compression dressing was applied, and a long knee brace was given for immobilization.

Rehabilitation

Postoperatively from the second day of surgery, patients were motivated to do static quadriceps, ankle pumps, gradual knee ROM, and straight leg raise and were allowed full weight-bearing ambulation using bilateral axillary crutch support with knee brace.

After two weeks, half squatting (closed kinetic exercises) and stair climbing with hinge knee cap and contralateral elbow crutch support were started. At three weeks, the brace was discarded, and walking without support was allowed. After six weeks, full squatting was started along with dynamic quadriceps using 2-5 kg weight, brisk walking, swimming, and eccentric quadriceps with a return to jogging at three months and continued till six months. The majority of patients returned to their previous occupation by six months, whereas heavy activities, such as running and sports, were allowed at nine months.

The patients were regularly followed up at intervals of two weeks, six weeks, 12 weeks, six months, and nine months. In the first visit, only local wound condition was handled, and ROM was evaluated. However, in all the subsequent visits, thorough clinical, radiological, and functional evaluation was carried out. Clinical examination included an anterior drawer test, and a radiological assessment was done with X-rays of the knee. Functional outcome was assessed using the Lysholm knee score.

Statistical analysis

IBM SPSS Statistics for Windows, Version 20.0 (Released 2011; IBM Corp., Armonk, New York, United States) was used for analysis. Continuous variables like age, surgery time, and height and weight measurement were expressed as mean±standard deviation, and intergroup comparison was followed across the four groups using the Mann-Whitney U test at 0.05 level of significance. The categorical variables like sex, side, and occupation were expressed as the number of patients, and the variable significance level was identified using Pearson's chi-squared test at 0.05 level of significance.

## Results

Age distribution

The age group of 21-40 years constituted about 68.9% of patients. The mean age in this study was 29.88±9.02 years. The youngest patient was 18 years old, and the oldest patient was 50 years old (Table 1).

**Table 1:** Age distribution

Age (years)	No. of patients	Percentage (%)	P-value
≤20	7	15.56	0.983
21-30	21	46.66	
31-40	10	22.22	
41-50	7	15.56	
Total	45	100	

Sex distribution

In this study, 39 (86.7%) patients were male and six (13.3%) patients were female.

Side of injury distribution

The right knee was affected in 26 (57.6%) patients which was almost comparable to the left knee in 19 (42.4%) patients.

Mode of injury distribution

**Figure 1:**
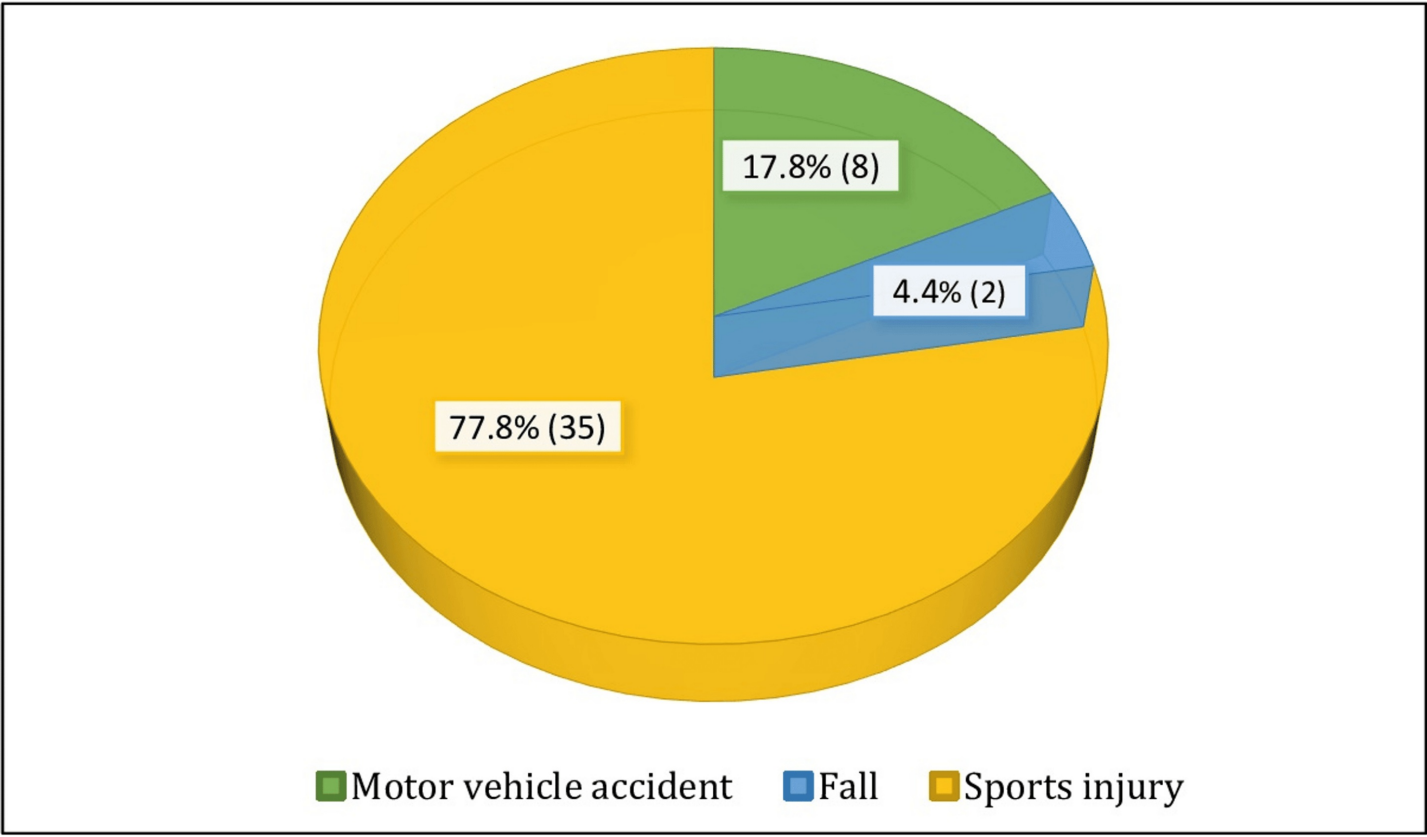
Mode of injury distribution

In this study, 35 (77.8%) patients sustained trauma during sports activities, eight (17.8%) were involved in motor vehicle accidents, and two (4.4%) suffered a fall (Figure 1).

Body mass index (BMI) distribution

Table 2 shows the mean values of patient parameters like height, weight, and BMI in our study group.

**Table 2:** BMI BMI: body mass index

	Mean	Standard deviation
Height (cm)	169.53	6.70
Weight (kg)	69.98	6.23
BMI	24.36	1.81

Occupation distribution

**Figure 2:**
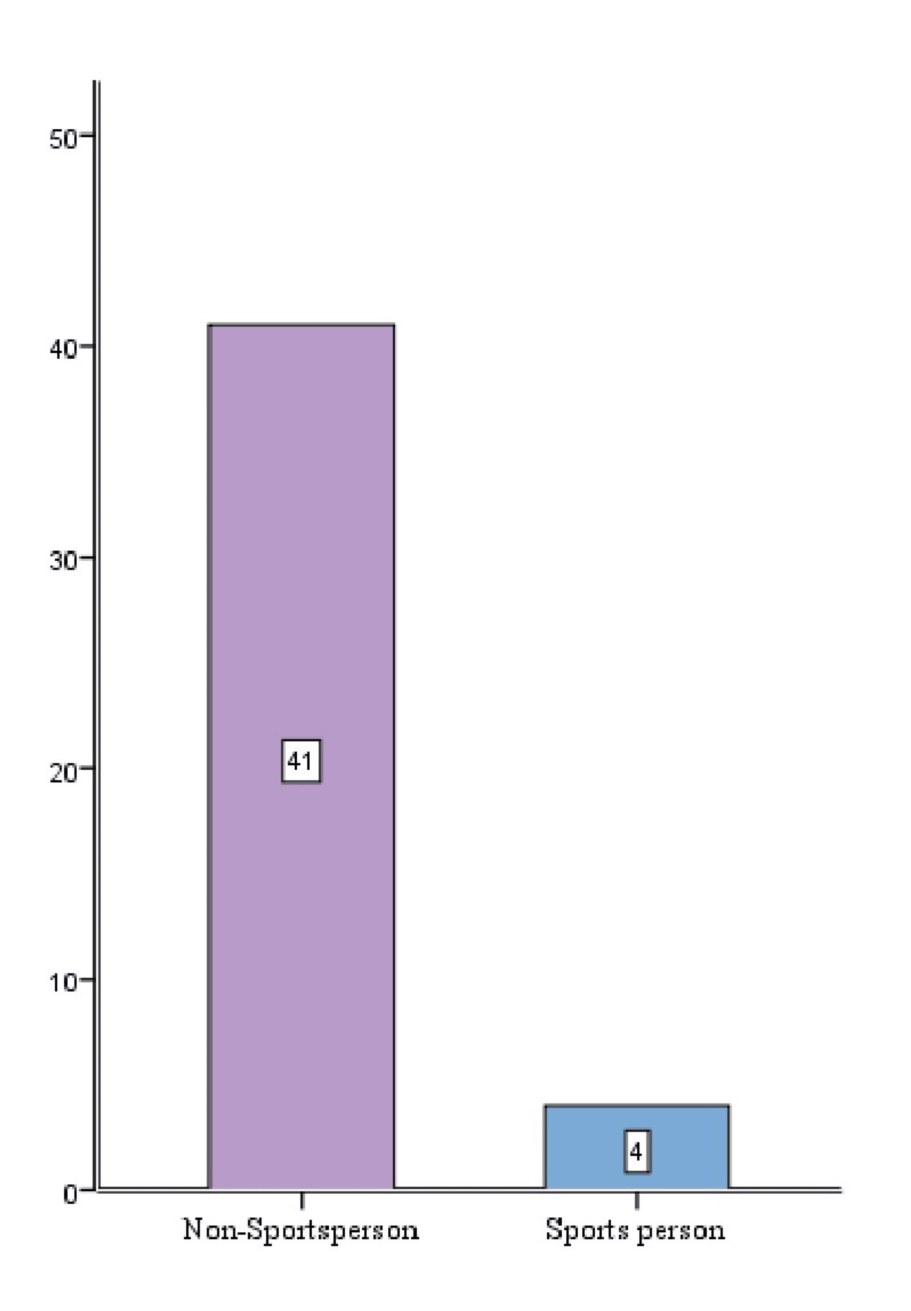
Occupation distribution

Figure 2 shows the distribution of occupation of the individuals in this study group. Non-sports persons (91.1%) were found to be more commonly affected than sports persons.

Preoperative clinical test for knee instability distribution

**Figure 3:**
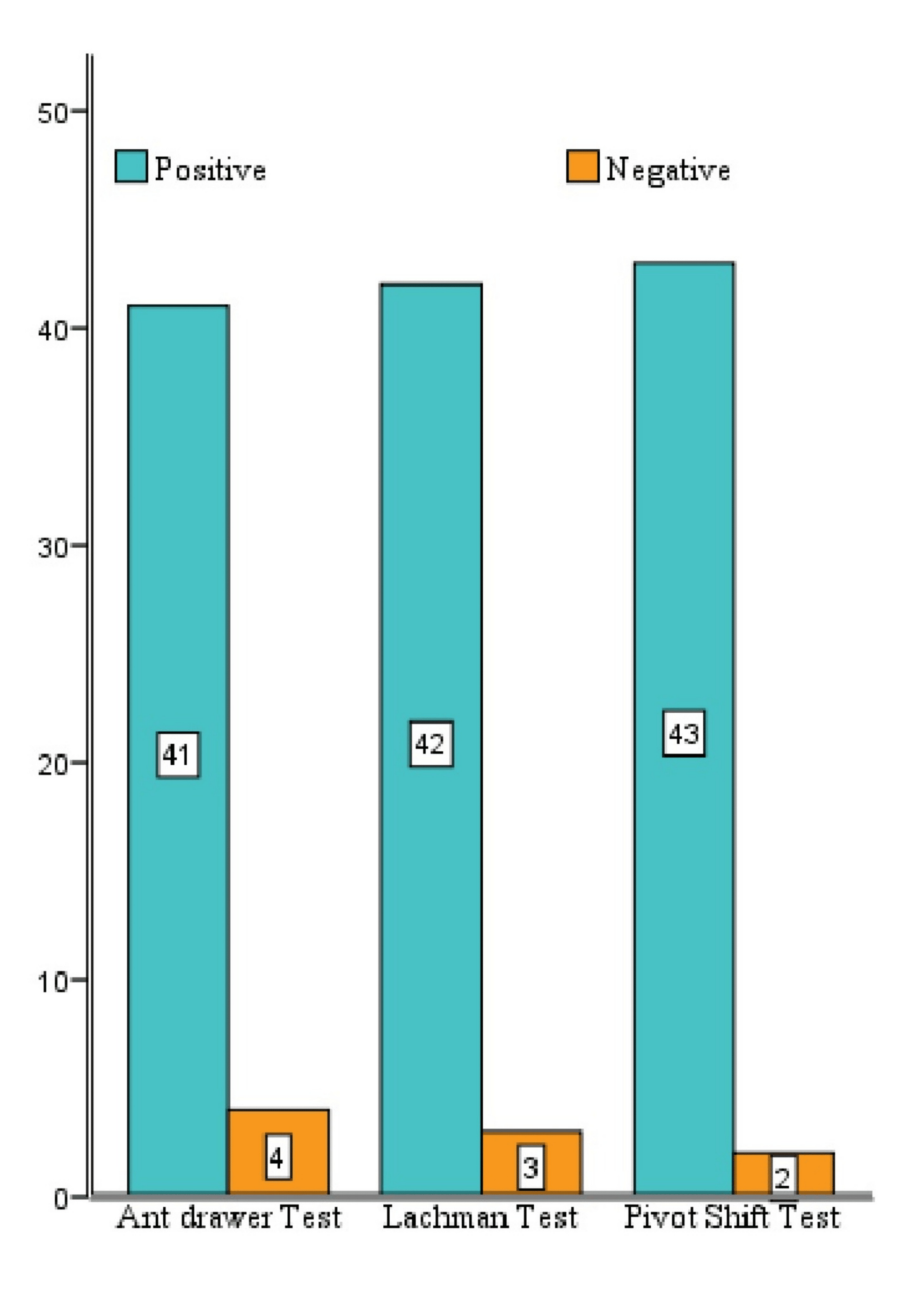
Clinical tests for knee instability

Figure 3 illustrates the distribution of preoperative clinical findings. About 91.1% demonstrated a positive anterior drawer test, 93.3% positive Lachman test, and 95.5% positive pivot shift test.

Pattern of ACL tear distribution

Table 3 shows the distribution of the pattern of ACL tears in this study. Femoral side tears were most common (77.8%) followed by mid-substance tears (17.8%), whereas tear from the tibial side was the least common (4.4%).

**Table 3:** Pattern of ACL tear ACL: anterior cruciate ligament

Tear pattern	No. of patients	Percentage (%)	P-value
Mid-substance	8	17.78	0.001
Femoral side	35	77.78	
Tibial	2	4.44	
Total	45	100	

Interval between injury and surgery

The average time elapsed between the time of injury and the time of surgery was 25.97±27.58 weeks (p=0.058).

Type of graft distribution

Table 4 reveals that in 95.6% of the patients, only semitendinosus graft was enough. Nevertheless, in 4.4% of cases, it needed to be supplemented by the gracilis graft also.

**Table 4:** Type of graft

Graft	No. of patients	Percentage (%)
Semitendinosus	43	95.56
Semitendinosus+gracilis	2	4.44
Total	45	100

ROM comparison

Table 5 elucidates that when compared with preoperative mean ROM, the postoperative ROM was increased (from 94.22° to 120.22°).

**Table 5:** ROM ROM: range of motion

ROM (degree)	Mean	Standard deviation	P-value
Preoperative	94.22	18.64	0.001
Postoperative	120.22	8.65	

Lysholm score

The mean Lysholm score for 45 patients was 95.31±6.55 by the end of nine months.

Lysholm score outcome

**Figure 4:**
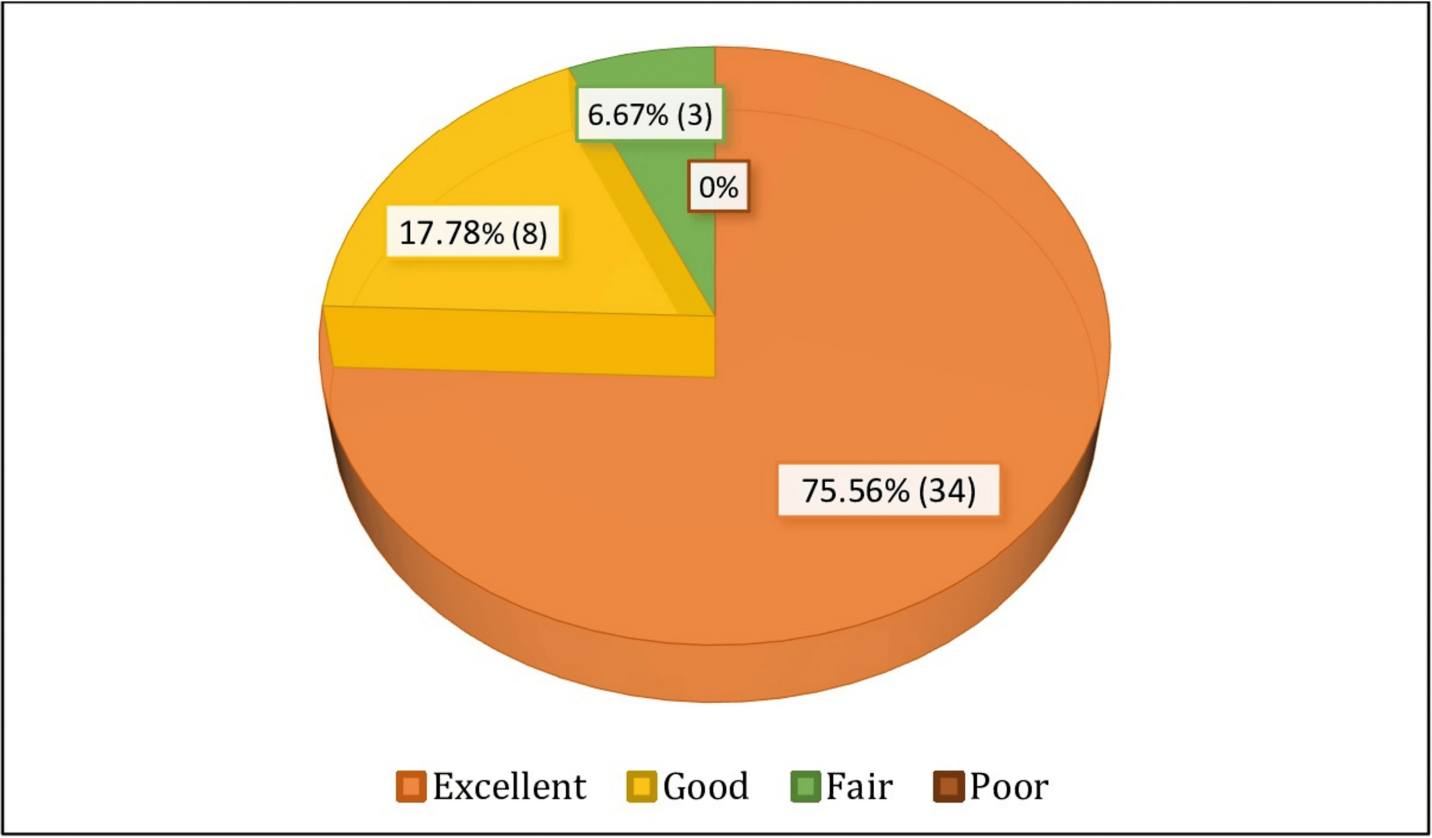
Lysholm score outcome

Thirty-four (75.56%) patients had excellent results, while eight (17.78%) patients demonstrated good results. Three (6.67%) patients showed a fair outcome, whereas no patient had a poor result at the final follow-up (Figure 4).

Complications

Forty-three (95.6%) patients did not have any complications. One patient developed a superficial skin infection (2.2%) in the form of serosanguinous discharge from the operative site which was treated by a short course of oral antibiotics. One patient (2.2%) reported knee stiffness which was believed to be due to non-compliance with the physiotherapy protocol.

None of the patients complained of anterior knee pain or patellofemoral crepitus at the final follow-up. Other complications such as persistent pain, arthrofibrosis, graft failure or rupture, or persistent joint instability were not seen. Revision surgery was not required for any patient.

## Discussion

The ACL is one of the most important structures for maintaining stability and is also one of the most commonly injured ligaments of the knee joint, particularly in people participating in high-contact sports like basketball, football, and skiing. People with ruptured ACL have functional instability and often increased anterior laxity, reduced activity and participation in sports, as well as increased risk of osteoarthritis in the long term as unstable knees generally get more damaged over time.

ACL reconstruction particularly in young and active patients allows for a return to sports activities and prevents inadvertent falls in day-to-day living. It provides appropriate stability to the knee and protects the knee from further cartilage damage, meniscal injuries, and osteoarthritis. Anatomic SB ACL reconstruction by arthroscopy has become the gold standard treatment for ACL tears, especially in young, active individuals. Biomechanical studies have demonstrated that anatomic SB ACL reconstructions restore knee kinematics to near-native states, comparable to DB reconstruction [16].

We did a comparative correlation with similar previous studies. Akoto and Hoeher [17] studied 88 patients (75 males and 13 females) whose mean age of presentation was 31 years (range 16-47 years). In a study done by Devgan et al. [18], 30 patients were studied with the mean age of presentation being 25 years, and all of them were males. Streich et al. [19] studied 40 patients, of which 28 were males and 12 were females with a median age of presentation of 29 years. In our study, 45 patients were taken, of which 39 were males and six were females and the mean age of the patients was 29.88 years. Young male persons in the Indian population are usually active and more commonly involved in sports activities and outdoor work which possibly is the reason behind making them prone to injuries.

Tan et al. [20] in a systematic review and meta-analysis on the importance of patient sex on ACL reconstruction found that there were comparable or inferior results in females compared to males in all the outcome parameters analyzed, i.e., the subjective and functional outcomes and the ability to return to sports. However, our study results did not correlate with the same. The functional outcome in females was found to be comparable to males. This may be possible due to the smaller sample size of the female population in our study.

The right knee was more commonly injured in our study, in 26 out of 45 patients, which was similar to the finding in the study by Devgan et al. [18]. However, no positive significant correlation was observed between the side of injury and functional outcome.

The mean BMI in our study was 24.36±1.81, and no correlation was noted between the BMI and the functional outcome. Kluczynski et al. [21] systematically reviewed seven studies that examined the effect of BMI on outcomes after ACL reconstruction. Four of these studies found an association between high BMI and worse outcome measures, and only three of these studies evaluated the association between complications and BMI but none observed significant findings.

In our study, the most common mode of injury was due to sports activities in 35 (77.8%) patients. However, we observed that the injury more commonly occurred in persons engaging in recreational sports (41 patients) compared to professional sports persons (four patients). The most common mechanism of injury is abduction flexion and internal rotation by the shift of the body weight on the fixed tibia. This finding was consistent with the findings obtained by Devgan et al. [18] and Streich et al. [19].

There remains debate in the literature regarding the optimal timing of surgery. Smith et al. [22] concluded from their systematic review that there were no statistically significant differences in outcome scores, patient satisfaction, return to play, laxity, ROM, arthrofibrosis, chondral injuries, patellofemoral pain, meniscal injuries, thromboembolic episodes, or need for revision surgery between early (<3 weeks) and delayed (>6 weeks) ACL reconstruction. In our study, the mean time from injury to ACL reconstruction was found to be 25.8 weeks, but we observed no correlation with the functional outcome at nine months follow-up which is consistent with the findings of the previous studies.

Regarding the limp, we found that from the preoperative scores, there was a significant improvement postoperatively and all the patients were free from limp except one at three months postoperatively. This was probably due to non-compliance with the rehabilitation protocol, but further at six months follow-up, after physical rehabilitation, he was limp-free. All the patients could mobilize at three months postoperatively without crutch support. Compared to preoperative locking scores, most of the patients were free of locking sensation or episodes at six weeks postoperatively.

As for instability, the mean instability score preoperatively was 10.11±4.88. In our study group, 66.7% of the patients had a feeling of giving way during routine activities, and 33.3% had a feeling of giving way during sports activities before surgery. Our findings were comparable with the findings by Devgan et al. [18] in which 73.3% (22) gave way during normal activities and 26.6% (eight) during sports activities. It significantly improved postoperatively with patients reporting no instability after six weeks and also at the final follow-up when they were allowed sports activities.

We documented the pain scores of the patients and found that there was significant improvement in pain at six weeks postoperative follow-up with all of the patients becoming pain-free by six months. As for swelling, there was no significant change between preoperative and six weeks postoperatively which could be attributed to further local soft tissue injury caused by the operative trauma and relative quadriceps wasting. The swelling majorly subsided within three to six months duration, and there was no swelling after six months in almost all the patients.

Squatting was also assessed preoperatively and postoperatively. Half squatting was allowed after three weeks, and full squatting was allowed at six weeks. There was a significant improvement in squatting scores by the end of nine months compared to the preoperative scores.

There is general consensus in the literature about anatomical ACL reconstruction being superior to non-anatomic ACL reconstruction. However, there is still an ongoing debate regarding SB and DB ACL reconstruction. The studies by Xu et al. [23] and Karikis et al. [24] have shown both modalities to be equally effective treatment options although DB ACL reconstruction has been reported to be associated with increased incidence of medial patellofemoral cartilage damage and poor posterolateral bundle status and long-term arthritis.

We evaluated the functional outcome using the Lysholm knee score. The mean Lysholm knee score increased from 49.28±13.78 preoperatively to 95.31±6.55 at nine months post-surgery. At the final follow-up, 93.33% of patients had scores in the range of 84-100 consistent with good to excellent functional outcomes. The study by Williams et al. [25] for a minimum two years follow-up following ACL reconstruction using hamstring autograft demonstrated the mean improvement in Lysholm knee score from 55 to 91 points. In a study by Streich et al. [19], the mean Lysholm score was 93.2±7.8 (range 66-100) at follow-up. Twenty-eight (70%) patients were rated as very good, nine (23%) as good, and three (7%) as fair in their study at 10 years follow-up. These data thus suggest that arthroscopic anatomical ACL reconstruction by SB technique using quadrupled hamstring autograft is an effective treatment modality with good outcomes.

Limitations of our study include the small number of patients, the absence of a control group, short-term follow-up, and bias due to a single hospital.

## Conclusions

Anatomical SB arthroscopic ACL reconstruction using hamstring autograft effectively restores the ligament to its native anatomical position. It is an effective treatment modality for ACL injuries. It leads to improvement in knee biomechanics and kinematics and also restores the stability of the knee in both anteroposterior and rotational planes. This technique is associated with satisfactory recovery of knee joint function, acceptable fixation, and a low number of complications and assures an early return to active lifestyle and sports activities. Aggressive and supervised physical therapy and rehabilitation which aids in gaining good functional stability and restoration of muscle strength is essential for achieving optimal functional outcomes following ACL reconstruction. Over the long run, it has produced excellent clinical, radiological, and functional outcomes without any long-drawn disability.
